# Clinical significance of EBP50 overexpression assessed by quantum dot analysis in gastric cancer

**DOI:** 10.3892/ol.2013.1271

**Published:** 2013-03-26

**Authors:** XIAO-GUANG LV, XIAO-FEI LEI, MENG-YAO JI, XU-FENG GUO, JING WANG, WEI-GUO DONG

**Affiliations:** Department of Gastroenterology, Renmin Hospital of Wuhan University, Wuhan, Hubei 430060, P.R. China

**Keywords:** ezrin-radixin-moesin-binding phosphoprotein 50, tumor marker, gastric cancer, quantum dot, prognosis, immunochemistry

## Abstract

Ezrin-radixin-moesin-binding phosphoprotein 50 (EBP50) is a postsynaptic density-95/disc-large/zonula occludens-1 (PDZ) homologous domain-containing protein that is involved in cell signaling. EBP50 regulates cell apoptosis, proliferation and invasion. In the present study, the prognostic impact factor of EBP50 expression was evaluated using a quantum dot (QD)-based assay and immunohistochemistry (IHC). The EBP50 protein expression in gastric cancer (GC) tissues was evaluated using IHC and QD-IHC. The study included 101 patients with GC (29 females and 72 males, aged 24–81 years), diagnosed and treated at the General Surgery Department of Renmin Hospital of Wuhan University (Wuhan, China) between 2000 and 2005. The survival rate was calculated using the Kaplan-Meier method and log-rank tests. IHC and QD analyses of 101 GC tissue specimens revealed that EBP50-positive tumor cells were frequently present in GC. Increased EBP50 immunostaining was observed in 63 specimens (62.4%). The EBP50 expression levels were correlated with increased tumor size and the male gender. EBP50 was well distributed in the cytoplasm and nuclei of the GC cells. However, EBP50 protein expression exhibited no correlation with age, differentiation, stage or lymph node metastasis. There were no associations between the expression of EBP50 and the mean survival rates (IHC, 50.5 vs. 58.1 months, P>0.05; QD, 55.4 vs. 63.2 months, P>0.05). These findings suggest that EBP50 protein expression is not correlated with the prognosis of patients with GC. QD-IHC and IHC have similar advantages for the detection of EBP50 protein expression.

## Introduction

Gastric cancer (GC) is the fourth most common type of cancer in the world. The incidence rates for GC are highest in males from Northeast Asia (Japan, Korea and China) (1), with up to 69 cases per 100,000 individuals per year. The carcinoembryonic antigen, CEA, and the carbohydrate antigens (CA) 19-9 and 72-4, have previously been used as tumor markers (2–3). Specifically, they have been used as reliable markers for monitoring tumor progression and the response to treatments for GC, including chemotherapy and radiation therapy (4). In the majority of patients with CA 19-9- and CA 72-4-positive GC, a decrease in these marker levels is correlated with a positive clinical outcome following successful resection and treatment (5). Consequently, these antigens are not so accurate as tumor progression predictors for GC diagnosis and follow-up. Therefore, it is important to develop additional markers for the screening and follow-up of patients with GC. Considerable efforts have been dedicated to the identification of sensitive and specific markers for GC (6–9). If the clinical predictors that identify patients with a low-risk of cancer recurrence following surgical resection were determined, then low-risk patients would be able to avoid unnecessary post-operative chemotherapy, thus improving their quality of life.

Ezrin-radixin-moesin-binding phosphoprotein 50 (EBP50) is a 358-amino acid protein containing two post-post-synaptic density-95/disc-large/zonula occludens-1 (PDZ) domains. EBP50 functions as a linker between membrane proteins and the cytoskeleton network and is involved in various types of cancer (10–11). EBP50 is important in cancer progression as it regulates cell proliferation and migration (12). Several studies have observed that EBP50 is a novel marker for various types of cancer, including breast cancer, and that EBP50 is able to predict the clinical behavior of these tumors (11). In a previous study, EBP50 was positively associated with tumor grade, prognosis and the estrogen receptor in the circulatory lymphocytes and breast cancer tissues (13).

EBP50 has been shown to be expressed in gastric parietal cells, as opposed to the mucous epithelium of the stomach which only expresses ezrin (14). However, to the best of our knowledge, there is no available data concerning EBP50 expression in GC. On the basis of data from previous cancer studies, we hypothesized that the EBP50 protein expression level was correlated with the progression of GC. Therefore, quantum dot (QD) and immunohistochemistry (IHC) assays were performed to investigate the prognostic value of EBP50 in GC.

## Materials and methods

### Patients

The present study included 101 GC patients (29 females and 72 males, aged 24–81 years and of Chinese nationality) diagnosed and treated at the General Surgery Department of Renmin Hospital of Wuhan University (Wuhan, China) between 2000 and 2005. Resected tissues were fixed in formalin, then embedded in paraffin. Stage IV GC tissue was unavailable due to a requirement for surgery in the affected patients (Table I). The tumor staging was based on a histopathological analysis and clinical assessment, according to the TNM (tumor-node-metastases) classification. Patients were staged according to the American Joint Committee on Cancer-International Union Against Cancer classification (15). For statistical analysis, the GC patients were divided into two groups, with 47 cancer patients in the stage I–II group and 54 patients in the stage III group. The patients were also subdivided into four groups depending on the degree of gastric wall invasion (T_1_, T_2_, T_3_ and T_4_) and four other groups depending on the nodal involvement (N_0_, N_1_, N_2_ and N_3_). The number of patients in these analyzed subgroups is shown in Table I. The present study was approved by the Ethics Committee of Wuhan University. Consent was received from all patients and all clinical investigations were performed according to the principles of the Declaration of Helsinki.

### IHC analysis

The GC tissues were fixed in 10% buffered formalin, embedded in paraffin and cut into 4-*μ*m sections. The sections were deparaffinized in xylene, rehydrated in a series of descending ethanol concentrations and incubated in 0.03% hydrogen peroxide for 10 min. Antigen retrieval was performed in 10 mM sodium citrate buffer (pH 6.0) for 15 min. The tissue sections were then incubated with an anti-EBP50 antibody (1:800 dilution, PA5-17045; Thermo Scientific, Rockford, IL, USA) at room temperature for 40 min. Following incubation, the specimens were washed with 0.5% Tween, 0.1 M Tris-base, 0.9% NaCl, (TBS-T; pH 7.6) and incubated with peroxidase- labeled polymer at room temperature for 30 min. The samples were then washed with TBS-T buffer and incubated with freshly prepared 3,3′-diaminobenzidine tetrahydrochloride (DAB) and substrate-chromogen buffer at room temperature for 8 min. Immunohistochemical reactions were developed in freshly prepared DAB (DAB kit; Fujian Maixin Biological Technology Ltd., Fujian, China) at room temperature for 8 min, then lightly counterstained with hematoxylin prior to mounting.

### QD fluorescence IHC

The specimen treatment by QD-IHC was similar to conventional IHC. The QD-IHC assay was performed according to the manufacturer’s instructions (Wuhan Jiayuan Quantum Dot Co., Ltd., Wuhan, China). Antigen retrieval was performed in citric acid (10 mM, pH 6.0) at 95°C for 10 min and the samples were cooled for 30 min. For the antibody binding, the specimens were first incubated in a 2% bovine serum albumin buffer (Sigma, St. Louis, MO, USA) at 37°C for 30 min and then at 4°C overnight with poly-rabbit anti-EBP50 antibodies (1:800 dilution, PA5-17045; Thermo Scientific, Rockford, IL, USA). The specimens were then washed three times with TBS-T for 5 min each wash and incubated in biotinylated goat anti-rabbit or anti-mouse IgG (1:100 dilution, Jackson ImmunoResearch, West Grove, PA, USA) at 37°C for 30 min.

For the QD conjugation, the antibody-bound specimens were incubated in 2% BSA buffer again at 37°C for 10 min, then incubated with QDs conjugated to streptavidin (QD-SA; 1:200 dilution in 2% BSA; Wuhan Jiayuan Quantum Dot Co., Ltd.) at 37°C for 30 min, washed three times with TBS-T for 5 min and finally sealed with 90% glycerin (Sigma).

The QD signal was detected with an Olympus BX51 fluorescence microscope equipped with an Olympus Micro DP 72 camera. The signal was red, target-specific, bright and photo-stable. The images for each specimen were analyzed using the WuDa Image Analysis System 2003, a multifunctional pathology analysis software package developed by two of the present study authors (16–18). For further quantification and statistical analysis, the numerical calculations for the two key variables in EBP50 detection, the fluorescence intensity and the distribution area, were based on spectral unmixing and the QDs were obtained as the final results.

### Scoring of QD-IHC and IHC

Using a 400X objective lens, ≥100 cells were randomly selected and counted from five representative fields of each core by two independent observers blinded to the samples identities. The scoring of the QD-IHC and IHC was based on the percentage of positive levels as follows: No staining or weak staining in <10% of the tumor cells (0); weak staining in >10% of the tumor cells (1+); complete staining of the membrane with weak or moderate intensity in >10% of the tumor cells (2+); and marked staining in >10% of the tumor cells (3+) (19).

### Statistical analysis

All statistical analysis was performed using SPSS software, Version 13.0 (SPSS Inc., Chicago, IL, USA). The differences between the two groups, based on the clinicopathological factors, were statistically analyzed using the Student’s t-test and the Chi-squared test. The survival rates were calculated according to the Kaplan-Meier method, using a log-rank test. P<0.05 was considered to indicate a statistically significant difference.

## Results

### Detection of EBP50 expression by IHC

**Figure 1:**
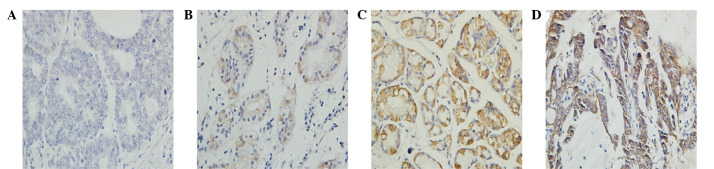
Immunohistochemistry showing EBP50 staining in GC. Sample of (A) negative EBP50 protein expression, (B) a positive case scoring 1+; (C) a positive case scoring 2+, and (D) a positive case scoring 3+ [magnification, ×400; objective lens; *Streptomyces* avidin-peroxidase connection (SP) method]. EBP50, ezrin-radixin-moesin-binding phosphoprotein 50; GC, gastric cancer.

The EBP50 protein was overexpressed in the majority of gastric carcinoma tissues (63/101, 62.4%), of these, 37 tissue samples (36.6%) were scored as 1+, 16 (15.8%) were scored as 2+ and 10 (9.9%) were scored as 3+ (Fig. 1). The expression of EBP50 detected by IHC was associated with tumor size and the male gender (P<0.05). The expression of EBP50 in the patients with GC increased with the tumor stage and was highest in the male patients (Table II).

### Detection of EBP50 expression by QD analysis

**Figure 2:**
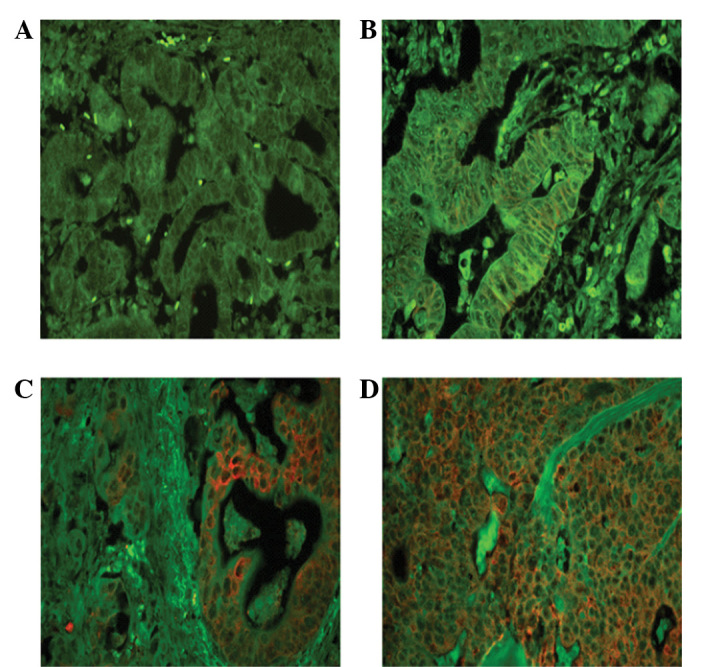
Analysis of EBP50 in advanced gastric cancer using quantum dots (QDs). (A) Sample negative for EBP50 protein expression; (B) a positive case scoring 1+; (C) a positive case scoring 2+; and (D) a positive case scoring 3+ (magnification, ×400; objective lens). EBP50, ezrin-radixin-moesin-binding phosphoprotein 50.

As shown in Fig. 2, the bright-red QD fluorescence specifically labeled tumor cells without nonspecific binding. The green background was from tissue autofluorescence. The score of the QDs was associated with the tumor size of the gastric carcinoma (P<0.05; Table II).

### Survival analysis

**Figure 3:**
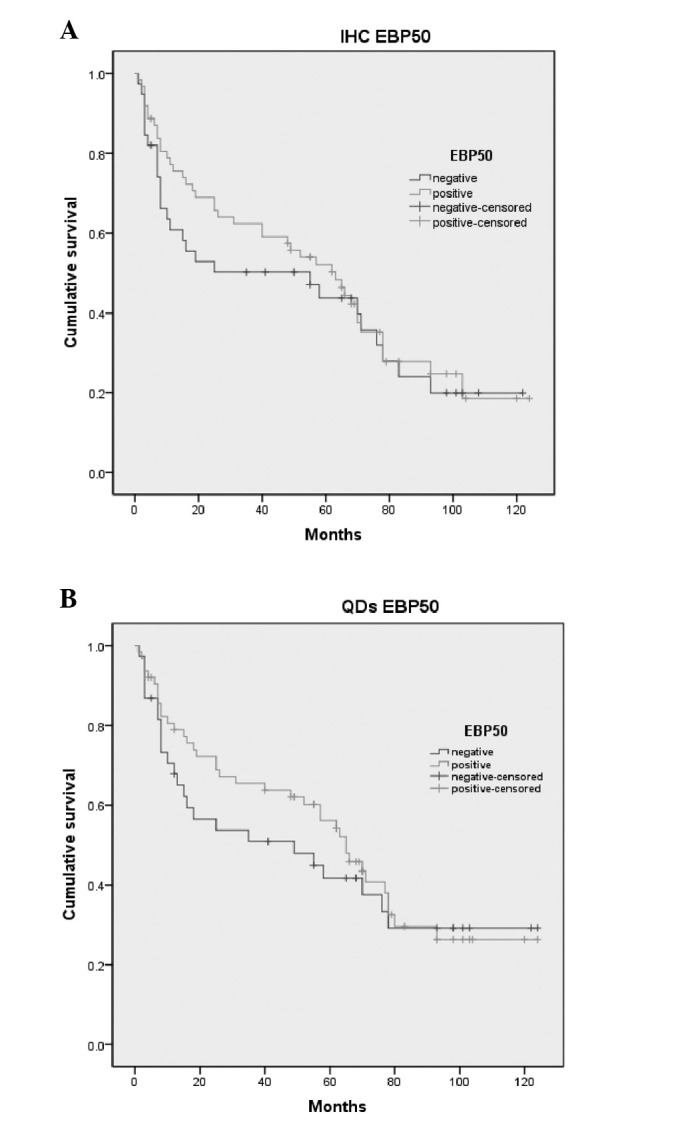
Kaplan-Meier plot for overall survival in 101 patients with advanced GC. The EBP50 protein expression was assessed using (A) IHC and (B) quantum dots (QDs). EBP50, ezrin-radixin-moesin-binding phosphoprotein 50; IHC, immunohistochemistry; GC, gastric cancer.

A survival analysis was performed on 101 patients who had survived for more than one month post-surgery. A total of 38 patients succumbed to GC within 124 months, while 63 had survived survived to January 1, 2012. The survival curves created according to the IHC and QD results of the EBP50 expression are shown in Fig. 3. The association between the expression of EBP50 and the mean survival rates was assessed by the Kaplan-Meier method. The survival rates with overexpression of EBP50 was higher than the negative one, however as P>0.05, there was no association between the survival rates and expression of EBP50. (IHC, 50.5 vs. 58.1 months, P>0.05; QD, 55.4 vs. 63.2 months, P>0.05; Fig. 3).

## Discussion

EBP50 may play an essential role in carcinogenesis, including that of breast cancer, colorectal cancer and hepatocellar carcinoma. The present study demonstrated that EBP50 is overexpressed in GC and that it is a novel marker of GC, as observed in previous studies (20–22). The expression of EBP50 was also observed to be correlated with the male gender and the tumor stage. In the present study, the bright red QD fluorescence specifically labeled the GC cell membranes without non-specific binding. Previous studies have demonstrated that EBP50 is important in cancer cell proliferation, invasion and metastasis. Therefore, we hypothesized that the EBP50 protein expression level may be correlated with the prognosis of GC. However, no significant correlation was observed between the expression of EBP50 and the overall survival rate of the GC patients. The observable differences in these studies may be a result of the usage of varying antibodies and scoring systems or, alternatively, may reflect the heterogeneity of GC between the various ethnicities. Gastric tumorigenesis is a multistep process that is initiated by benign and atypical hyperproliferation, is established as *in situ* carcinoma, progresses into invasive carcinoma and culminates in metastatic disease (1,20–21). However, the progression from *in situ* to invasive carcinoma is poorly understood. It is important to identify additional promising tumor markers to improve screening strategies for GC. In the present study, EBP50 immunoreactivity was significantly associated with the male gender and tumor invasion (T stage). These results suggest that EBP50 expression is associated with several malignant clinicopathological features of GC, although it is not a valuable predictor for the prognosis of GC patients.

Immunofluorescence labeling is a standard technique that is widely used in the biomedical field for the detection of biological macromolecules in tissue sections. However, at present, the available fluorescent labels are not stable and become irreversibly photobleached under high-intensity illumination. We used a validated QD-IHC protocol for quantifying EBP50 expression in formalin-fixed, paraffin- embedded specimens, which provided an accurate, sensitive and convenient approach. In the present study, 605-nm QD-SA conjugated probes were used for the detection of EBP50 expression. These probes have several advantages that are prerequisites for clinical application (22,23). First, the probes are more stable than QDs conjugated to antibodies. Second, the biotin-avidin staining system is commonly used in molecular pathology and is highly sensitive, therefore, QD-SA probes may be incorporated into the current detection system easily and conveniently.

In conclusion, the overexpression of EBP50 is correlated with the male gender and with tumor size in GC, but not with the patient survival time. Additionally, QD-IHC is as good as IHC for the detection of tumor markers when considering the accuracy of detecting the protein location, the photostability and the duration of the fluorescence lifetime.

## Figures and Tables

**Table I:** Characteristics of the patients with GC.

Tested group	Number (%)
Age, years	
≤50	24 (23.8)
>50	77 (76.2)
Gender	
Male	72 (71.3)
Female	29 (28.7)
Differentiation	
Well	16 (15.8)
Moderate	37 (36.6)
Undifferentiated	48 (47.6)
Tumor size	
T_1_	8 (7.9)
T_2_	21 (20.8)
T_3_	55 (54.5)
T_4_	17 (16.8)
Nodal metastasis	
N_0_	33 (32.7)
N_1_	48 (47.5)
N_2_	13 (12.9)
N_3_	7 (6.9)
Stage	
I–III	47 (46.1)
III	54 (53.9)

GC, gastric cancer.

**Table II:** Correlations between EBP50 protein expression and the clinical significance of 101 cases of GC.

	IHC assessment of EBP50				QD score of EBP50		
	
Tested group	Negative	Positive	Total	P-value	Number	QD (X±SD)	P-value
Age, years							
≤50	10	14	24	0.640	24	11.810±3.937	0.456
>50	28	49	77		77	13.250±3.407	
Gender							
Male	21	51	72	0.006	72	13.477±6.355	0.933
Female	17	12	29		29	9.433±5.687	
Differentiation							
Well	6	10	16	0.916	16	26.446±6.675	0.148
Moderate	13	24	37		37	11.633±4.846	
Undifferentiated	19	29	48		48	36.135±11.039	
Tumor size							
T_1_	6	2	8	0.020	8	33.708±17.851	0.029
T_2_	9	12	21		21	6.548±4.192	
T_3_	21	34	55		55	28.210±7.202	
T_4_	2	15	17		17	41.935±8.167	
Nodal metastasis							
N_0_	18	15	33	0.099	33	28.747±4.021	0.717
N_1_	15	33	48		48	20.057±8.102	
N_2_	3	10	13		13	29.271±10.734	
N_3_	2	5	7		7	25.645±15.087	
Stage							
I–III	35	12	47	0.537	47	28.467±9.127	0.918
III	43	11	54		54	25.941±15.540	

EBP50, ezrin-radixin-moesin-binding phosphoprotein 50; GC, gastric cancer; QD, quantum dot; IHC, immunohistochemistry.
